# Therapeutic Potential of Extracellular Vesicles for Demyelinating Diseases; Challenges and Opportunities

**DOI:** 10.3389/fnmol.2018.00434

**Published:** 2018-11-23

**Authors:** Iñaki Osorio-Querejeta, Ainhoa Alberro, Maider Muñoz-Culla, Imre Mäger, David Otaegui

**Affiliations:** ^1^Multiple Sclerosis Unit, Biodonostia Health Research Institute, San Sebastian, Spain; ^2^Spanish Network of Multiple Sclerosis, Barcelona, Spain; ^3^Department of Physiology, Anatomy and Genetics, University of Oxford, Oxford, United Kingdom; ^4^Institute of Technology, University of Tartu, Tartu, Estonia

**Keywords:** remyelination, exosomes, myelin, multiple sclerosis, microRNAs, EAE, oligodendrocyte

## Abstract

Multiple Sclerosis is a demyelinating disease of the central nervous system for which no remyelination therapy is available and alternative strategies are being tested. Extracellular vesicles (EVs) have emerged as players in physiological and pathological processes and are being proposed as therapeutic targets and mediators. More concretely, EVs have shown to be involved in myelination related processes such as axon-oligodendrocyte communication or oligodendrocyte precursor cell migration. In addition, EVs have been shown to carry genetic material and small compounds, and to be able to cross the Blood Brain Barrier. This scenario led scientists to test the ability of EVs as myelin regeneration promoters in demyelinating diseases. In this review we will address the use of EVs as remyelination promoters and the challenges and opportunities of this therapy will be discussed.

## Introduction

Myelin is a membranous sheath produced by oligodendrocytes (Ols) in the central nervous system (CNS) that surrounds axons allowing the saltatory nerve impulse transmission. Moreover, myelin protects axons and contributes to the maintenance of its homeostasis. Myelin can be damaged in a physiological context, due to the normal aging process, but it can also be caused by pathological mechanisms. The latter scenario occurs in a wide variety of pathological situations, such as traumatic demyelination, leukodystrophies or multiple sclerosis (MS), being the last one the most common demyelinating disease. Although no specific auto-antigen has been identified yet, MS is considered a chronic autoimmune CNS disease that includes the breakdown of the Blood-Brain Barrier, inflammation, demyelination, oligodendrocyte loss, gliosis and axonal degeneration (Baecher-Allan et al., 2018). It is accepted that the activation of peripheral autoreactive effector CD4^+^ T cells that migrate into the CNS attacking the myelin sheath is the main cause of MS appearance. Once in the CNS a second reactivation occurs in which other cells types such as B and CD8^+^ T cells of the adaptive immune response, together with natural killers and microglia cells of the innate immune system contribute to the disease causing oligodendrocyte destruction, myelin loss, and an imbalance of the homeostasis of axons (reviewed in Baecher-Allan et al., 2018). This imbalance causes axon damage and an inefficient nerve impulse transmission. In the first stages of the disease, myelin can be restored, recovering normal electrical signal transmission. This is a complex process named remyelination in which a dynamic combination of different signaling pathways and molecules such as growth factors, cytokines and chemokines are tightly regulated (Kuhlmann et al., 2008). To achieve remyelination, oligodendrocyte precursor cells (OPCs) need to (1) proliferate, (2) migrate into the lesions, and (3) differentiate to myelinating Ols that will generate new myelin (Miron et al., 2011). Nevertheless, with the progression of the disease this process tends to fail. It is not completely understood why remyelination capacity decreases with time, but a lack of OPCs, a poor migration of these cells or their impossibility to differentiate to Ols have been proposed (Franklin, 2002). In addition, it is increasingly recognized that age is not only a risk factor for neurodegeneration but also adversely influences regenerative processes and remyelination (Hampton et al., 2012). Moreover, some factors such as genetic background and diet are also involved in the reduction of the remyelination capacity (revised in Adamo, 2014).

To avoid neurodegeneration and promote neuroprotection, as well as the restoration of the fast saltatory conduction, the generation of new myelin is of extreme importance. The promotion of remyelination might protect axons avoiding their degeneration and probably improving patients’ prognosis. Therefore, pharmaceutical companies and researchers that work in the field are trying their best to develop new remyelination therapies.

To this end, the replacement of the endogenous OPC population and the stimulation of endogenous OPCs to regenerate myelin are being analyzed, being differentiation a key point in this process (Hartley et al., 2014). Finally, targeting the immune system has been also pointed out as a therapeutic strategy to induce remyelination (Dombrowski et al., 2017; El Behi et al., 2017).

An ideal therapy should be able to cross the BBB and reach the CNS, target OPCs and not other cell types and should have minimal side effects. Owing to their natural capacity to affect cell proliferation and differentiation, and their potential to cross BBB, extracellular vesicles (EVs) have emerged as highly promising candidates for the treatment of demyelinating diseases, as discussed in detail in the following sections.

## Why Extracellular Vesicles?

Intercellular communication is a key factor for the functioning and regulation of all biological processes. Apart from the two classical mechanisms – direct cell-to-cell communication and transfer of secreted soluble molecules –, in the last years extracellular vesicles (EVs) have been found to play a central role in intercellular communication.

Extracellular vesicles are membrane-bound particles secreted by cells. There are different types of EVs and the most common classification is based on their size and biogenesis (Raposo and Stoorvogel, 2013). EVs formed inside multivesicular bodies and released upon fusion of these bodies with the plasma membrane are called exosomes. Their main characteristic is to have a uniform size of between 30 and 150 nm, thus being the smallest EVs. On the other hand, those known as microvesicles, come from the evagination and direct budding from the plasma membrane. Microvesicles vary greatly in size, ranging generally from 0.3 to 1 μm in diameter; however, it must be noted that in many scenarios it can be difficult to separate exosomes from microvesicles purely based on their size (Willms et al., 2018). Another type of membrane vesicles are apoptotic bodies, which are 1–5 μm in size and were described many years ago and have different features to those derived from living cells (György et al., 2011). Currently, the generic term EV is used to refer to the complete set of secreted vesicles (Gould and Raposo, 2013). EVs play an essential role in indirect intercellular communication as their membrane, cytosolic proteins, lipids, metabolites and genetic material can be transferred between cells (Théry et al., 2001; Valadi et al., 2007). They can follow two different ways of integration: by direct fusion with the plasma membrane or by endocytosis (Morelli et al., 2004; Montecalvo et al., 2012).

Most cell types release EVs being secreted both in physiologic and pathogenic conditions. They can be isolated from many body fluids, including plasma and cerebrospinal fluid (CSF). EVs are involved in many biological processes, their capacity to regulate immune response and cell differentiation being the two most important processes in the context of this review (Robbins and Morelli, 2014). Moreover, EVs take part in the transmission of information across the CNS (Frühbeis et al., 2013b) and have been found to play a role in the regulation of synaptic activity (Fauré et al., 2006) and myelin sheath biogenesis (Marzesco et al., 2005; Bakhti et al., 2011), as well as in the repair of damaged neurons (Court et al., 2011).

The pathogenesis of several diseases has been shown to be linked to EVs, including cancer (Robbins and Morelli, 2014), neurodegenerative diseases (Basso and Bonetto, 2016; Thompson et al., 2016) and, of particular interest to this work, MS (Verderio et al., 2012; Sáenz-Cuesta et al., 2014a,b; Selmaj et al., 2017). The implication of EVs and their ability to carry messages from one cell to another suggests that the use of EVs as a drug delivery system or as a treatment, might be an interesting way of targeting and modulating the course of the disease. Moreover, the fact that EVs are able to cross the BBB makes them strong candidates for CNS disease therapy (Jan et al., 2017).

## Therapeutic Potential of Evs for Demyelinating Diseases

Several works have been published demonstrating the therapeutic potential of EVs. These works will be discussed in the following paragraphs and have been summarized in Table 1.

**Table 1:** Summary of therapeutic potential of EVs for demyelinating diseases.

Reference	EVs type	EVs Source	Isolation method	Principal experiment	Route of administration	Result
Williams et al., 2013	Exosomes	Virgin and pregnant mice serum	Ultracentrifugation	EAE	Intravenous	Stablished EAE supression.
Rajan et al., 2016	Exosomes	HPLSC culture supernatant	ExoQuick TC	EAE	Intravenous	Inmmunomodulation of EAE.
Zhuang et al., 2011	Exosomes	Glioblastoma culture supernatant	Sequential centrifugation steps	EAE	Intranasal	EAE inhibition.
Frühbeis et al., 2013a	Exosomes	Oli-Neu cultures supernatant	Sequential centrifugation steps	Oligodendrocyte-neuron co-culture	N/A	Exosomes mediated communication.
Krämer-Albers et al., 2007	Exosomes	Primary oligodendrocytes culture supernatant	Ultracentrifugation	Oligodendrocyte culture	N/A	Exosomes contain PLP, MBP, MOG and CNP.
Bakhti et al., 2011	Exosomes	Primary oligodendrocytes culture supernatant	Sequential centrifugation steps	Oligodendrocyte culture	N/A	Oligodendrocytes derived exosomes inhibit OPC differentiation.
Kurachi et al., 2016	Extracellular vesicles	MVECs culture supernatant	ExoQuick TC	Oligodendrocyte Precursor cell culture	N/A	OPCs survival, proliferation and motility.
Otero-Ortega et al., 2017	Exosomes	MSC culture supernatant	miRCURY Exosomes Isolation Kit	Subcortical ischemic stroke	Intravenous	Promotion of olifodendrocyte formation and remyelination.
Pusic and Kraig, 2014	Exosomes	Youth and Environmental Enriched rat serum	ExoQuick TC	Old rats	Intranasal	Enhanced myelin content.
Pusic et al., 2016	Exosomes	Environmental Enriched rat serum	ExoQuick TC	Demyeliantion hipocampal slice culture	N/A	Myelination increased and oxidative stress reduced.
Doeppner et al., 2015	Extracellular vesicles	MSC culture supernatant	PEG precipitation method	Ischemic stroke	Intravenous	Neuroprotection and neuroregeneration.
Drommelschmidt et al., 2017	Extracellular vesicles	MSC culture supernatant	PEG precipitation method	Perinatal brain induced inflammation	Intraperitoneal	Immunomodulation and reduction of micro- and astrogliosis.

In some demyelinating pathologies, such as MS, the immune system is responsible for the damage caused to myelin. In fact, all the available treatments for MS are immunomodulatory or immunoregulatory drugs that prevent autoimmune attacks on the myelin sheath. In this way, the ability of exosomes isolated from pregnant mice serum or human periodontal ligament stem cells-derived exosomes to reduce the clinical score of the Experimental Autoimmune Encephalomyelitis (EAE), an animal model of MS, has been addressed by inhibiting the immune response, and more concretely by dampening Th1 response (Williams et al., 2013; Rajan et al., 2016). In addition, the intranasal administration of curcumin-loaded glioblastoma-derived exosomes to EAE animals ameliorated the clinical symptoms of the model. Although the mechanism of action is not clear, the induction of immune tolerance and the apoptosis of activated immune cells are postulated to be behind this process. This data demonstrate that exosomes could work as anti-inflammatory drug delivery vehicles (Zhuang et al., 2011).

As was mentioned in the introduction, Ols are responsible for generating myelin that enwraps axons. The communication between Ols and axons is essential for the survival and functional maintenance of both. Interestingly, this communication between Ols and axons has been shown to be mediated by exosomes and, in addition, the interactions between Ols and axons might affect the cargo of exosomes (Frühbeis et al., 2013a). Moreover, when the cargo of exosomes released by Ols was analyzed, researchers found that those vesicles contained high levels of myelin related proteins; more concretely PLP, MBP, MOG, and CNP (Krämer-Albers et al., 2007). This data was the first evidence of the possible role that exosomes could be playing in myelination. In a more recent work, it was suggested that Ol-derived exosomes were able to inhibit the differentiation of OPCs (Bakhti et al., 2011). Even though the authors did not demonstrate the mechanism by which Ols regulate OPCs in an inhibitory way, these results reinforce the implication of exosomes in OPC differentiation, an essential step for myelination and remyelination. In a different work, the ability of pregnant mice serum-derived exosomes to promote the trafficking of OPCs into lesions from EAE mice after intravenous administration was shown (Williams et al., 2013) emphasizing the implication of exosomes in myelination related processes.

To analyze the role that EVs play in pathological systems, several models have been used. In a model of white matter infarction in rats, researchers demonstrated that EVs derived from microvascular endothelial cells (MVECs) were taken up by OPCs, inhibiting the apoptosis of OPCs and promoting survival, proliferation and motility of the cells. The authors demonstrate that those EVs contained microRNAs and adhesion molecules which were responsible for the shown effects (Kurachi et al., 2016). Moreover, Mesenchymal Stem Cell-derived exosomes (MSC-Exs) have been shown to promote oligodendrocyte formation and remyelination in a model of subcortical ischemic stroke. After intravenous administration of MSC-Exs, authors were able to detect higher levels of MOG protein and more myelinated axons. Interestingly, the 2416 proteins detected in the exosomes and described to be involved in brain repair functions were suggested by the authors as mediators of the effect (Otero-Ortega et al., 2017).

Furthermore, a work published in 2014 demonstrated that exosomes from young and environmentally enriched rats significantly increased the myelin content, oligodendrocyte precursor and neuronal stem cell levels and reduced oxidative stress and astrogliosis in demyelinated hippocampal slice cultures (Pusic and Kraig, 2014; Pusic et al., 2016). They also tested the effect of these exosomes *in vivo* by intranasal administration in aged rats, showing positive results in myelin generation. The authors related the exosome-derived pro-remyelination effect to their cargo, suggesting that the presence of miR-219 was responsible for promoting remyelination (Pusic and Kraig, 2014).

Another aspect of demyelinating diseases is that the lack of myelin wrapping axons might, if remyelination does not take place, induce the disruption of the axons and, therefore, neurodegeneration. Neuroprotection is a key factor which might improve patients’ outcome and increase their life quality. Regarding to this, mesenchymal stem cells derived EVs were shown to be effective peripheral immunomodulators in models of traumatic brain injury after both intravenous or intraperitoneal administration, decreasing inflammation and increasing neuroprotection, angiogenesis and neurological function, opening therapeutic possibilities in which neuroprotection can be reinforced (Doeppner et al., 2015; Drommelschmidt et al., 2017).

## Delivery Into the Central Nervous System

To be able to use EVs as therapeutic biopharmaceuticals for treating MS, it is imperative to ensure that EVs will reach their target cells in the CNS. That can be achieved, for example, by delivering EVs directly to the brain, by using systemic injections, or by administering vesicles via intranasal route. The intranasal route can be efficient for different cell type derived EVs, including T-cell, fibroblast and tumor derived exosomes (Zhuang et al., 2011). This delivery route not only leads to increased brain accumulation of exosomes, but more importantly, it also results in reduced inflammation in EAE animals if exosomes are loaded with therapeutic anti-inflammatory molecules, as was previously mentioned (Zhuang et al., 2011). The latter clearly underlines the potential of EVs for treating MS via the intranasal route, which is further supported by successful experiments conducted in the context of other CNS diseases such as Parkinson’s disease (PD). In a mouse model of PD, catalase-loaded macrophage exosomes reached the brain and provided antioxidant-mediated neuroprotection (Haney et al., 2015). Neuroprotection was also induced by curcumin loaded embryonic stem cell exosomes in an ischemia-reperfusion injury model (Kalani et al., 2016). Repeated treatments with curcumin loaded exosomes led to a reduction of inflammation and improved neurological score and restored the expression of several BBB proteins.

However, it appears that EV loading with exogenous cargoes prior to intranasal administration is not always essential for therapeutic effects in the CNS, as recently demonstrated in a status epilepticus mouse model. Unmodified human bone marrow derived MSC-Exs reduced neuron loss and inflammation in the hippocampus of treated mice, which more importantly led to preservation of memory function (Long et al., 2017). These properties of unmodified MSC-Exs for treating CNS disease are particularly interesting and promising for MS. Given the trend toward replacing certain MSC cell therapies with EV based therapies, and the fact that a number of MSC cell therapies have been tested in Phase I/II clinical trials for treating MS as well (Heldring et al., 2015), it is likely that MSC EVs will gain further focus in the short term for targeting MS pathology as well.

In addition to the intranasal administration route, as described above, other local delivery options have shown efficacy for EV based CNS therapies as well. Unilateral direct brain infusion of glioblastoma derived exosomes, pre-loaded with hydrophobic siRNA, led to exosome-dependent bilateral Huntington mRNA silencing in the brain of treated mice (Didiot et al., 2016). Other therapeutic strategies not directly relying on drug delivery can be efficient as well. Intracerebral neuroblastoma exosome administration to an Alzheimer disease mouse model reduced amyloid-β levels in the brain and lowered the associated synaptotoxicity, tapping thus into natural EV-mediated Aβ clearance pathways (Yuyama et al., 2014). Similar effects were observed also when using primary neuron exosomes, the effect being cell type specific as glial exosomes were less efficient in the capture of amyloid-β (Yuyama et al., 2015). This is not surprising as the transport of exosomes to brain parenchyma can be specifically related to the presence of specific surface molecules such as folate receptor α (Grapp et al., 2013) as well as other EV related signatures that can, for example, mediate periphery-brain signaling in inflammation (Balusu et al., 2016).

In many cases, however, systemic rather than local therapeutic EV administration would be preferred for various reasons, including the safety of the treatment administration. Despite the fact that BBB is virtually impermeable to most molecules there is some evidence that unmodified exosomes can enter the brain to some extent (Yang et al., 2015), but brain exposure is significantly increased when using certain brain targeting ligands such as the rabies glycoprotein derived RVG peptide (Wiklander et al., 2015). The brain targeting RVG peptide, even though the precise targeting mechanism has not been fully elucidated, led to increased brain delivery of siRNA when decorated on dendritic cell exosomes (Alvarez-Erviti et al., 2011). Using that strategy, it was possible to lower the levels of Bace1 on both mRNA and protein levels in the brains of wild type mice (Alvarez-Erviti et al., 2011), and in reduced level of α-synuclein mRNA in S129D α-Syn transgenic mice (Cooper et al., 2014).

## Challenges and Opportunities

**FIGURE 1:**
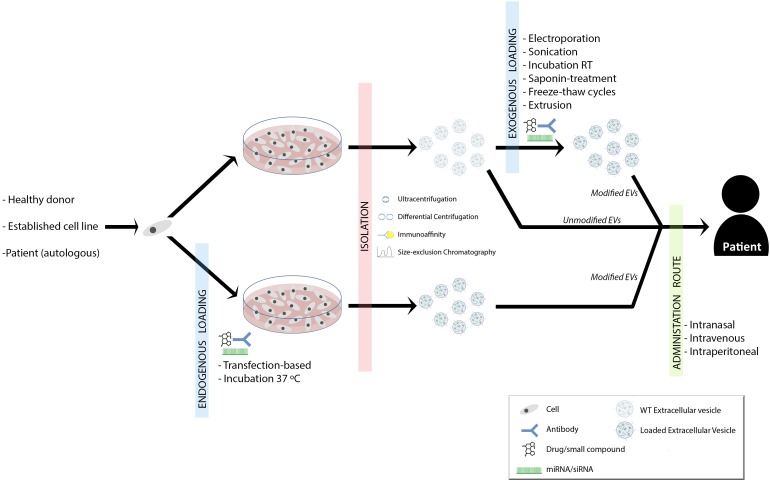
Summary of proposed therapeutic approaches for demyelinating diseases. Established cell lines, donor- or patient-derived cells are isolated and grown. EVs can be loaded with drugs/small compounds, miRNAs/siRNAs and/or surface antibodies, which provide new options in remyelination therapy. The loading can be performed during the cell culture (endogenous loading) or once EVs are isolated (exogenous loading∖reviewed in Vader et al., 2016). This might depend on the strategy and purpose of the therapy (Sutaria et al., 2017). Therapeutic EVs can be isolated by ultracentrifugation, differential centrifugation, immunoaffinity or size-exclusion chromatography (Lener et al., 2015). Finally, EVs could be administered to the patient intranasally, intraperitoneally or intravenously and again, this is something that will vary according to the therapeutic strategy.

Two characteristic aspects of MS are inflammation and neurodegeneration. The inhibition of inflammation and the promotion of remyelination are postulated as two therapeutic ways to improve patients’ outcome. As it has been widely shown, EVs can play a role in both immunomodulation and remyelination. But, what is the future going to be like with EVs mediated MS therapy? (Figure 1).

### The First Thing to Consider

The first thing to consider is the source where EVs are isolated from. In this sense established cells lines or cell isolated from the patient or a compatible donor can be used to isolate EVs. Biofluids such as plasma or urine are also an alternative. It is not clear which source is the most efficient and each one has got detractors. In this sense and as explained above, several are the sources that have been used with promising results, opening a wide range of EVs origins to be used. However, we consider that EVs isolated from cell culture might be more reproducible and “easy to manage.” In this way, cell therapies derived EVs are suggested as strong candidates as disease treatment. The use of cell-free stem cell-based therapy decreases the risk of cell therapy maintaining the beneficial effect of those cells. As an example, Mesenchymal Stem Cell derived vesicles have been widely studied as therapeutic mediators for several diseases (Review in Börger et al., 2017; Phinney and Pittenger, 2017). We consider that it might be a feasible treatment for MS acting as immunomodulatory agents and tissue repair mediator. In addition to the source, the isolation method is also a relevant aspect to be mentioned, as several methods can lead to different EVs types. Although several effectors has been made in order to standardize isolation techniques, there is still controversy (Gardiner et al., 2016).

### A Second Consideration

A second consideration can be the use of non-modified or bioengineered vesicles. The use of non-modified EVs has shown promising results (Pusic and Kraig, 2014; Pusic et al., 2014). In fact, there are several clinical trials recruiting patients in which the ability of allogenic mesenchymal stem cell derived exosomes in acute ischemic stroke or the effect of plasma derived exosomes on cutaneous wound healing will be addressed (NCT02565264, 2015; NCT03384433, 2017). Nevertheless, the modification of the cargo of EVs by bioengineering techniques is an interesting and promising field in EV-mediated therapies and we consider that it might be a more effective treatment method. It has been proved that cells which are genetically modified to overexpress a concrete microRNA, release EVs enriched in that microRNA (Squadrito et al., 2014). In this sense, microRNAs have shown to be involved in the differentiation of OPCs; more concretely miR-138, miR-219 and miR-338 (Dugas et al., 2010; de Faria et al., 2012; Wang et al., 2017). The enrichment of those microRNAs in the cargo of EVs might induce OPC differentiation and therefore remyelination after demyelination. Vesicles can also be loaded with small compounds and drugs with anti-inflammatory effects. In this sense, curcumin loaded exosomes demonstrated to induce neuroprotection (Kalani et al., 2016). We also propose that nowadays immunomodulatory drugs could also be loaded in exosomes in order to obtain a controlled and direct administration into the CNS. This therapeutic approach is of interest due to the immunological component of MS. Finally, EVs can be modified to express membrane receptors of the target cell, in this way increasing the uptake by the cell and decreasing non-specific bindings (Alvarez-Erviti et al., 2011).

Extracellular vesicles have demonstrated that they are key players in myelin regeneration and the applications that EVs could have in the stimulation of remyelination in pathological states are many. As we have mentioned previously, treatment to induce remyelination is still not available and the use of EVs is becoming a promising and feasible method to immunomodulate, induce myelin restoration, and in this way decreasing neurodegeneration and therefore, increasing patients’ outcome. However, even if the implication of EVs in remyelination related processes has been addressed in several works, our knowledge about the therapeutic potential of EVs is just beginning and an exciting future is awaiting us.

## Author Contributions

IO-Q wrote the sections “Introduction and Therapeutic Potential of EVs for Demyelinating Diseases” and had produced the table and the figure. AA wrote the section “Why Extracellular Vesicles?” IM wrote the section “Delivery into the Central Nervous System.” MM-C and DO supervised the work. All authors contributed to the section “Challenges and Opportunities.”

## Conflict of Interest Statement

The authors declare that the research was conducted in the absence of any commercial or financial relationships that could be construed as a potential conflict of interest.
